# The carbon footprint of the perioperative transurethral resection of bladder tumour pathway

**DOI:** 10.1111/bju.16477

**Published:** 2024-07-25

**Authors:** Joseph B. John, Michael Collins, Sophie Eames, Kieran O’Flynn, Tim W.R. Briggs, William K. Gray, John S. McGrath

**Affiliations:** ^1^ University of Exeter Medical School Exeter UK; ^2^ Department of Urology Royal Devon University Healthcare NHS Foundation Trust Exeter UK; ^3^ Getting it Right First Time Programme NHS England London UK; ^4^ Environmental Resources Management London UK; ^5^ Department of Surgery Royal National Orthopaedic Hospital Stanmore, London UK; ^6^ Department of Urology, Salford Royal Northern Care Alliance NHS Foundation Trust Salford UK

**Keywords:** environmental impact, sustainability, sustainable surgery, bladder cancer, surgery

## Abstract

**Objectives:**

To evaluate the carbon footprint of the perioperative transurethral resection of bladder tumour (TURBT) pathway from decision to treat to postoperative discharge, and model potential greenhouse gas (GHG) emissions reduction strategies.

**Materials and Methods:**

This process‐based attributional cradle‐to‐grave life‐cycle assessment (LCA) of GHG emissions modelled the perioperative TURBT pathway at a hospital in Southwest England. We included travel, energy and water use, all reusable and consumable items, and laundry and equipment sterilisation. Resource use for 30 patients undergoing surgery was recorded to understand average GHG emissions and the inter‐case variability. Sensitivity analysis was performed for manufacturing location, pharmaceutical manufacturing carbon‐intensity, and theatre list utilisation.

**Results:**

The median (interquartile range) perioperative TURBT carbon footprint was 131.8 (119.8–153.6) kg of carbon dioxide equivalent. Major pathway categories contributing to GHG emissions were surgical equipment (22.2%), travel (18.6%), gas and electricity (13.3%), and anaesthesia/drugs and associated adjuncts (27.0%), primarily due to consumable items and processes. Readily modifiable GHG emissions hotspots included patient travel for preoperative assessment, glove use, catheter use, irrigation delivery and extraction, and mitomycin C disposal. GHG emissions were higher for those admitted as inpatients after surgery.

**Conclusions:**

This cradle‐to‐grave LCA found multiple modifiable GHG emissions hotspots. Key mitigation themes include minimising avoidable patient travel, rationalising equipment use, optimally filling operating theatre lists, and safely avoiding postoperative catheterisation and hospital admission where possible. A crucial next step is to design and deliver an implementation strategy for the environmentally sustainable changes demonstrated herein.

**Abbreviations**
BEIS: UK Department for Business Energy and Industrial Strategy
CHP: combined heat and power
DEFRA: UK Department for Environment, Food and Rural Affairs
EPR: electronic patient records
GHG: greenhouse gas
HSDU: hospital sterilisation and decontamination unit
kgCO_2_e: kg of carbon dioxide equivalent
LCA: life‐cycle assessment
LDU: laundry unit
POAC: preoperative assessment clinic
PPE: personal protective equipment
TURBT: transurethral resection of bladder tumour

## Introduction

There is increasing acknowledgement that we must reduce healthcare related greenhouse gas (GHG) emissions as we seek to avert the worst effects of the evolving climate crisis [1, 2, 3, 4]. An important part of this approach involves generating data insights to inform us about where modifiable GHG emissions hotspots exist in the care pathways that we deliver for patients. Selective use of cradle‐to‐grave life‐cycle assessment (LCA) methodology can be a useful means of achieving this [5].

Operating theatres produce the greatest GHG emissions of any part of the hospital [6]. Whilst there is a growing body of evidence informing us about particularly environmentally harmful elements of surgical care, no studies have assessed the delivery of a perioperative endourology pathway as a means of identifying hotspots and directing mitigation [7].

### Objectives

- To evaluate the carbon footprint of the perioperative transurethral resection of bladder tumour (TURBT) pathway from decision to treat to postoperative discharge.
- To model potential GHG emissions reduction strategies.

## Methods

This is a process‐based cradle‐to‐grave LCA of GHG emissions for the perioperative pathway for patients undergoing TURBT. A comprehensive inventory of materials and energy flows was constructed and real‐world utilisation of inventory resource was measured for patients over 30 cases (Appendix S1: Table S1). This analysis was performed at a single hospital trust in Southwest England over 8 months during 2022–2023. Operations in two different theatre suites (dedicated day‐case unit and main theatres) were recorded.

### Case Inclusion Criteria

Any patients undergoing TURBT for a new or recurrent bladder tumour.

### Case Exclusion Criteria

Cystoscopic management of bladder tumour not amounting to resection, i.e. cystoscopy and diathermy ablation ± biopsy.

### Functional Unit and Perioperative Pathway Definition

The functional unit was the perioperative TURBT pathway for one patient. This was defined as beginning with the patient as an outpatient following a decision to treat a newly identified primary or recurrent bladder tumour and ending with the patient arrival home after discharge from hospital following surgery. Pathway exclusions were the episode at which the bladder tumour was identified (i.e. flexible cystoscopy or imaging), and subsequent follow‐up care after the patient had travelled from hospital following their TURBT.

The GHG emissions are presented as carbon dioxide equivalents (CO_2_e) expressed in terms of Global Warming Potential over 100 years (GWP100).

[Correction added on 13 August 2024, after first online publication: The preceding subsection header has been corrected and the contents have been updated.]

### Boundary Inclusions

- Patient and staff travel.
- Gas and electricity use.
- Water supply and treatment.
- Life cycle of single‐use items (raw materials, manufacture, transport, disposal).
- Life cycle of reusable items (raw materials, manufacture, transport, reprocessing, disposal).
- Direct care environments: preoperative assessment clinic (POAC), elective surgical admissions ward, operating theatres and recovery, inpatient ward.
- Furniture, fixtures, and fittings used in direct care environments.
- Pathway service environments; laundry unit (LDU), hospital sterilisation and decontamination unit (HSDU).

### Boundary Exclusions

- Capital goods used in pathway service environments (LDU, HSDU), and on‐site electricity generation, heating, ventilation, and air‐conditioning, due to an anticipated negligible contribution to per‐case GHG emissions.
- Air conditioning refrigerant use, due to variability in use according to season and geography.
- Maintenance of capital equipment, due to practical limitations in characterising this for a very large inventory of materials.
- Histology processing, due to a small carbon footprint identified by Gordon et al. [8].

Figure S1 outlines the system boundaries.

### Data Sources and Assumptions

#### Patient and Staff Travel

Data from a 2021 commissioned trust travel census of 822 staff members and 60 patients was used. The average round trip journey distance for staff and patients was calculated using this data, and weight adjusted for the proportions of different modes of transport used. Emissions factors for different modes of transport were sourced from The UK Department for Environment, Food and Rural Affairs (DEFRA) and Department for Business, Energy and Industrial Strategy (BEIS) GHG Conversion Factors for Company Reporting database (2022) [9]. The travel census was assumed to be representative for all staff, and for patients attending POAC. For patients attending for surgery, all were assumed to have been driven by car due to the need to undergo regional or general anaesthetic.

A staff inventory was produced for each care environment and pathway service environment, and the attributable per‐patient number of staff members for each environment calculated. This meant that staff travel‐related GHG emissions could be attributed to each patient's case. Staff inventory and travel data are shown in Tables S3 and S4 in Appendix S1, respectively.

#### Gas, Electricity

We used trust energy consumption data from April 2021 to March 2022 inclusive. During this period the main hospital site generated 65% of its electricity from an on‐site combined heat and power (CHP) gas‐powered generator. The National Grid supplied 33% of electricity, and on‐site solar energy and backup diesel generation both contributed 1%. The annual use of gas and electricity attributable to the urology service and one urology operating theatre was determined by multiplying overall energy use by the fractional hospital area used to deliver operative care or a direct support service. No specific submeter readings were available for hospital areas within the boundary conditions. The energy consumption figures were then divided by the approximate number of patients receiving the service from each different hospital area annually, to achieve per‐patient attributable gas and electricity use. This annual patient number was modelled based on an assumption that all TURBT operations were of average length. These calculations and underlying assumptions are outlined in Appendix S1, Tables S5.a–d (electricity) and S6.a,b (gas).

#### Water

Water use and disposal data from April 2021 to March 2022 (inclusive) for the main hospital was obtained and water consumption apportioned according to relative floor area and patient volume. Direct water use measurements were not recorded based on low estimated GHG emissions contribution from materiality assessment. The trust‐wide return to sewer rate was 83%. These were used when determining GHG emissions due to wastewater. DEFRA/BEIS emissions factors for water supply and treatment were used. Table S7.a,b in Appendix S1 outline the calculation steps, underlying assumptions.

#### Decontamination and Sterilisation

All reusable items processed through HSDU were identified within the inventory. The resource used for each step of reprocessing of each item or set was determined. This included manual decontamination, machine decontamination, autoclave sterilisation, packaging, and personal protective equipment (PPE) use. Gas, electricity, water and steam use were determined based on manufacturer product specifications. Steam was generated using a gas boiler that is separate to the CHP generator. Gas powered boiler model and product specification details were unavailable and therefore the boiler was assumed to have the same characteristics to the Fulton J series described by Rizan et al. [10]. The GHG emissions per unit mass of steam used to heat the machine decontaminator and autoclave were derived from the Fulton's published product specification.

Manual decontamination was estimated to take 10 min/load, using 25 L of water heated by 30° Kelvin. It was estimated in consultation with HSDU staff that one manual decontamination load (and set of PPE) could accommodate all reusable items reprocessed by HSDU for one TURBT. GHG emissions were estimated on a per‐set and a per‐item basis, based on an optimal filling estimate for the machine decontaminator and autoclave steriliser. The calculation steps and assumptions are outlined in Table S8.a,b. Separate to the energy utilised during decontamination and sterilisation, HSDU building gas and electricity consumption was estimated to be the same as the elective surgical admissions ward, which is similarly sized.

### Laundry

The LDU is a large unit located on‐site, which also serves numerous hospitals across Southwest England. The LDU monitor use of gas, electricity, water and fuel oil, and data covering April 2020–March 2022 inclusive were obtained. Detergent use is also monitored, and average weekly use over 13 weeks between November 2022 and June 2023 was acquired. GHG emissions due to degradation of detergents in wastewater was also estimated [11]. A mass adjusted estimate of the GHG emissions due to laundering different linen items was produced. LDU modelling is the same as described by John et al. [12].

### Clinical Environments

An inventory of all materials and processes was obtained for all clinical environments in which patients undergoing TURBT were managed. A combination of direct observation and use of electronic patient records (EPR) was used to establish resource use. EPR were used to determine administered medications and the number of clinical observations taken for each patient.

### Equipment and Consumables

Following materiality assessment, inventory items and their packaging were weighed. The mass of large items that could not be weighed was obtained from open‐source data or from manufacturers directly. Where this was not possible, equipment masses were estimated. The material composition of consumables was identified whenever possible. For complex equipment the relative proportion of composite materials was estimated. The mass of single‐use equipment packaging was obtained. The estimated lifespan of reusable items is outlined in the inventory.

The Neptune suction device (Stryker Corp., Kalamazoo, MI, USA) was introduced to the urology operating theatre part way through this study. Stryker have performed their own LCA of this device, and their inventory of materials was shared with us to facilitate our own estimates for the Neptune's per‐use GHG emissions. This ensured methodological consistency within our study. In‐use electricity consumption was not accounted for, with a static ‘average case’ estimate used for cases where the Neptune was deployed. The full inventory of materials for the Neptune is available on request from Stryker corp.

### Anaesthetic Gas

The average mass of sevoflurane gas delivered for five cases was determined by weighing the vaporisers in the anaesthetic room and operating theatre before and after each case. The average sevoflurane use calculated was then assumed to be representative of all cases involving sevoflurane use.

### Waste

The mass of waste according to different waste streams was determined according to how each product was observed to be disposed of. For incinerated items, the product's contained carbon was used to calculate CO_2_ emissions from incineration. It was assumed that no additional fuel was required for incineration. DEFRA/BIES transport emissions factors were used to estimate the GHG emissions due to transporting waste. The GHG emissions due to processing recycled waste was considered out of scope, in keeping with the GHG protocol guidance [13].

### Emissions Factors

The emissions factors used are listed in Table S2. This includes factors that were derived during our modelling. Appropriate emissions factors were applied to account for the contained materials within products, and GHG emissions associated with their processing from extraction as a raw material through to disposal. Emissions factors were mostly sourced from EcoInvent V3.10 (2023, cutoff by classification, IPCC 2021), the BEIS/DEFRA database (2022), and factors published in academic journals [9, 14, 15, 16, 17, 18]. Biogenic carbon was accounted for in the case of organically derived products such as cotton, cardboard, and paper.

### Patient Level Data Collection

After the inventory was produced, resource use for the care of 30 patients undergoing TURBT was recorded between December 2022 and July 2023. We used purposive sampling to obtain data on the practice of six urologists and nine anaesthetists until data saturation of observed practice was reached, with a variety of different tumour sizes treated. Basic demographic data and information describing tumour size was also recorded.

### Data Analysis

Descriptive analysis was performed, and data explored to identify GHG emissions hotspots within the pathway. Sensitivity analysis was performed for key assumptions, and brief scenario modelling performed for potential areas of GHG mitigation. All inventory items were categorised and subcategorised for the purpose of hotspot identification. For subcategory assignment, sevoflurane, propofol and local anaesthetic used in spinal anaesthesia were labelled as ‘anaesthetic agents’. All other pharmaceutical products including mitomycin C were subcategorised as ‘drugs’. Data were managed, analysed and modelled using a series of custom databases and an author‐built calculator in Microsoft Excel (Microsoft 365; Microsoft Corp., Redmond, WA, USA).

## Results

**Fig. 1:**
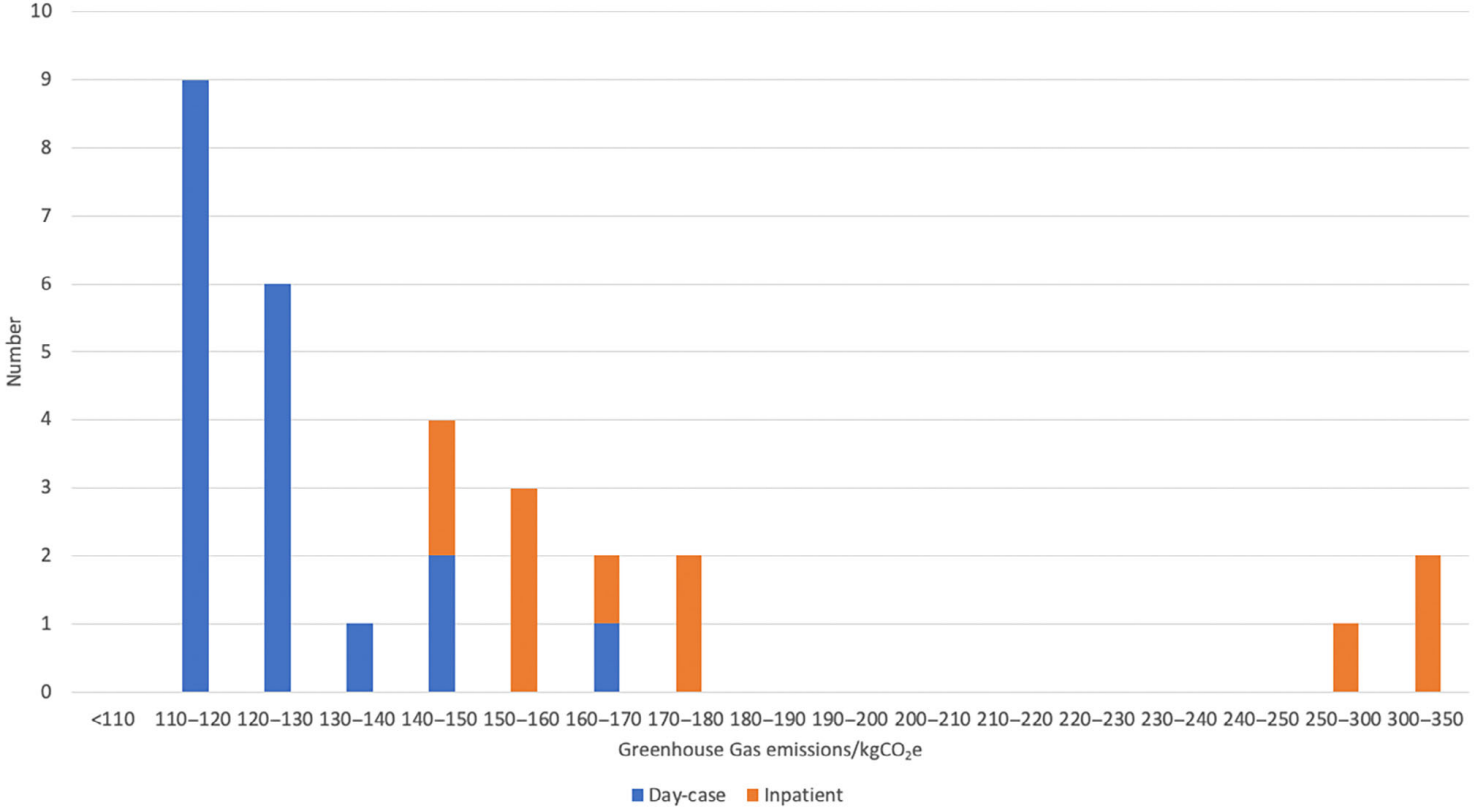
Distribution of total GHG emissions for 30 TURBT perioperative pathways.

Table 1 summarises patient and tumour characteristics, and anaesthetic choice. The median patient age was 80 years and 63% of operations were delivered as a day case. The median (interquartile range; range) GHG emissions for the 30 cases was 131.8 (119.8–153.6; 115.2–347.3) kg of carbon dioxide equivalent (kgCO_2_e). The mean (sd) GHG emission was 153.8 (58.9) kgCO_2_e. This is equivalent to driving 713 km in an average sized car – further than the distance from London to Edinburgh or from Paris to Toulouse. Figure 1 shows the distribution of total case GHG emissions for each patient and specifies whether each case was a day case or inpatient operation. The median day case and inpatient TURBT involved 121.3 and 161.9 kgCO_2_e of attributable GHG emissions, respectively.

**Table 1:** Patient, tumour, and operative characteristics of patients undergoing TURBT.

Characteristic	Value
Age, years, median (IQR; range)	80 (72–84; 50–90)
Male sex, *n* (%)	21 (70)
ASA grade, *n* (%)	
1	0 (0)
2	12 (40)
3	17 (57)
4	1 (3)
LOS (nights in hospital), *n* (%)	
0	19 (63)
1	8 (27)
2	1 (3)
5	2 (7)
Anaesthetic, *n* (%)	
Inhalation anaesthesia	18 (60)
TIVA	11 (37)
Spinal	1 (3)
Tumour grade and stage, *n* (%)	
Benign	6 (20)
Squamous metaplasia	1 (3)
Low/int grade NMIBC	5 (17)
High grade NMIBC/CIS	13 (43)
MIBC	5 (17)
Mass resected, g, median (IQR; range)	2.25 (1–7.25; 0.8–70)

ASA, American Association of Anaesthesiology; CIS, carcinoma *in situ*; IQR, interquartile range; MIBC, muscle‐invasive bladder cancer; NMIBC, non‐muscle‐invasive bladder cancer; TIVA, total intravenous anaesthesia.

**Fig. 2:**
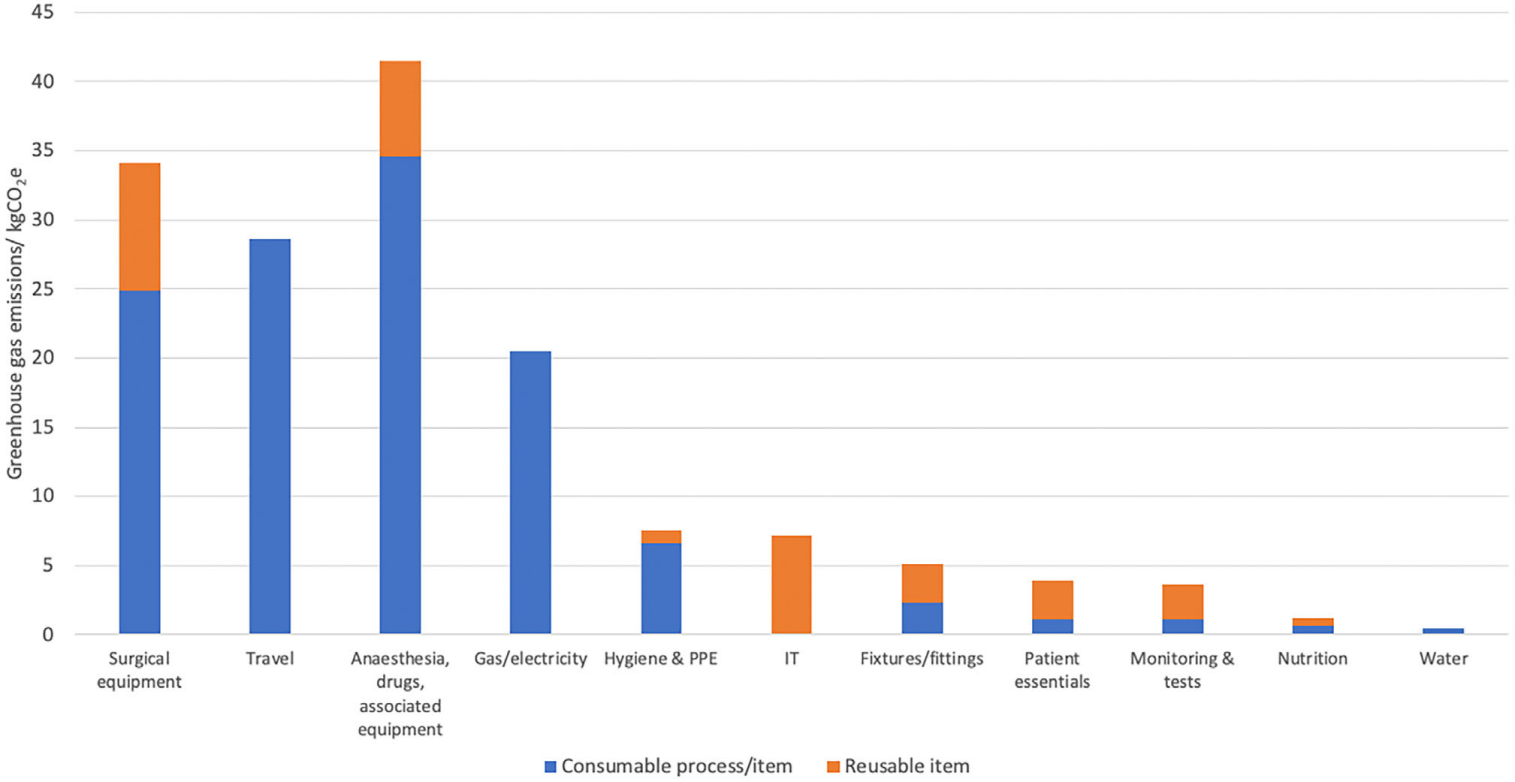
Mean GHG emissions by category for the perioperative TURBT pathway.

Figure 2 outlines the mean GHG emissions according to different pathway categories. Surgical equipment, travel, gas/electricity, and anaesthesia, drugs and associated equipment comprise 22.2%, 18.6%, 13.3% and 27.0% of the pathway emissions, respectively. Figure 2 also delineates consumable items and processes from reusable ones. The former category includes GHG emissions due to single‐use items, fuel use, and consumable chemicals and pharmaceuticals.

**Fig. 3:**
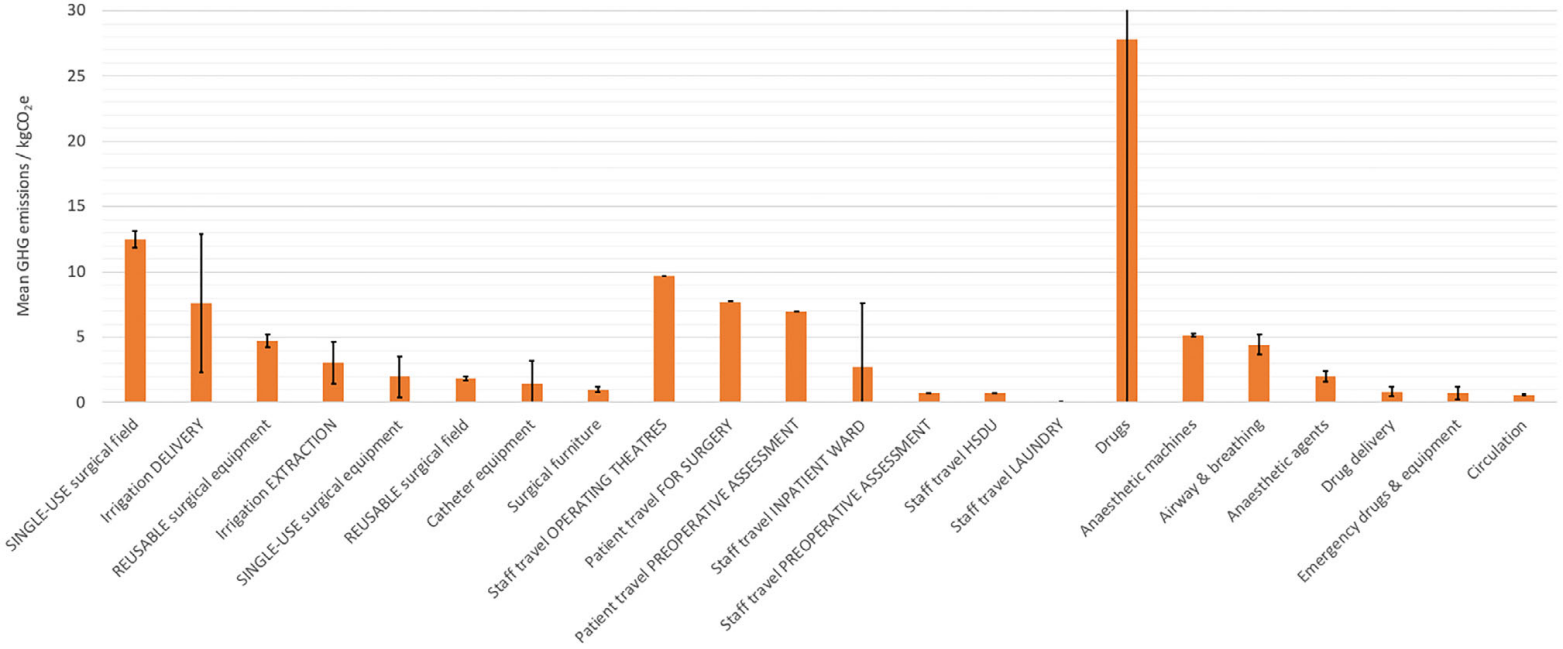
Mean GHG emissions by subcategory for the perioperative TURBT pathway.

Figure 3 shows GHG emissions associated with subcategories of the largest emissions sources: surgical equipment, travel, anaesthesia, drugs, and associated equipment. The sd for each of these categories is also shown. The largest subcategories are drugs, staff travel to operating theatres, single‐use surgical field equipment, patient travel for surgery, patient travel for preoperative assessment, and irrigation delivery.

Figure S2.a,b (Appendix S1) are heat maps showing the hotspots for surgical equipment at a patient level. This identifies areas with high associated GHG emissions for particular patients, which are not necessarily illustrated in mean values. Hotspot areas for certain patients are irrigation solution, mitomycin C disposal, irrigation disposal, and catheter equipment.

**Fig. 4:**
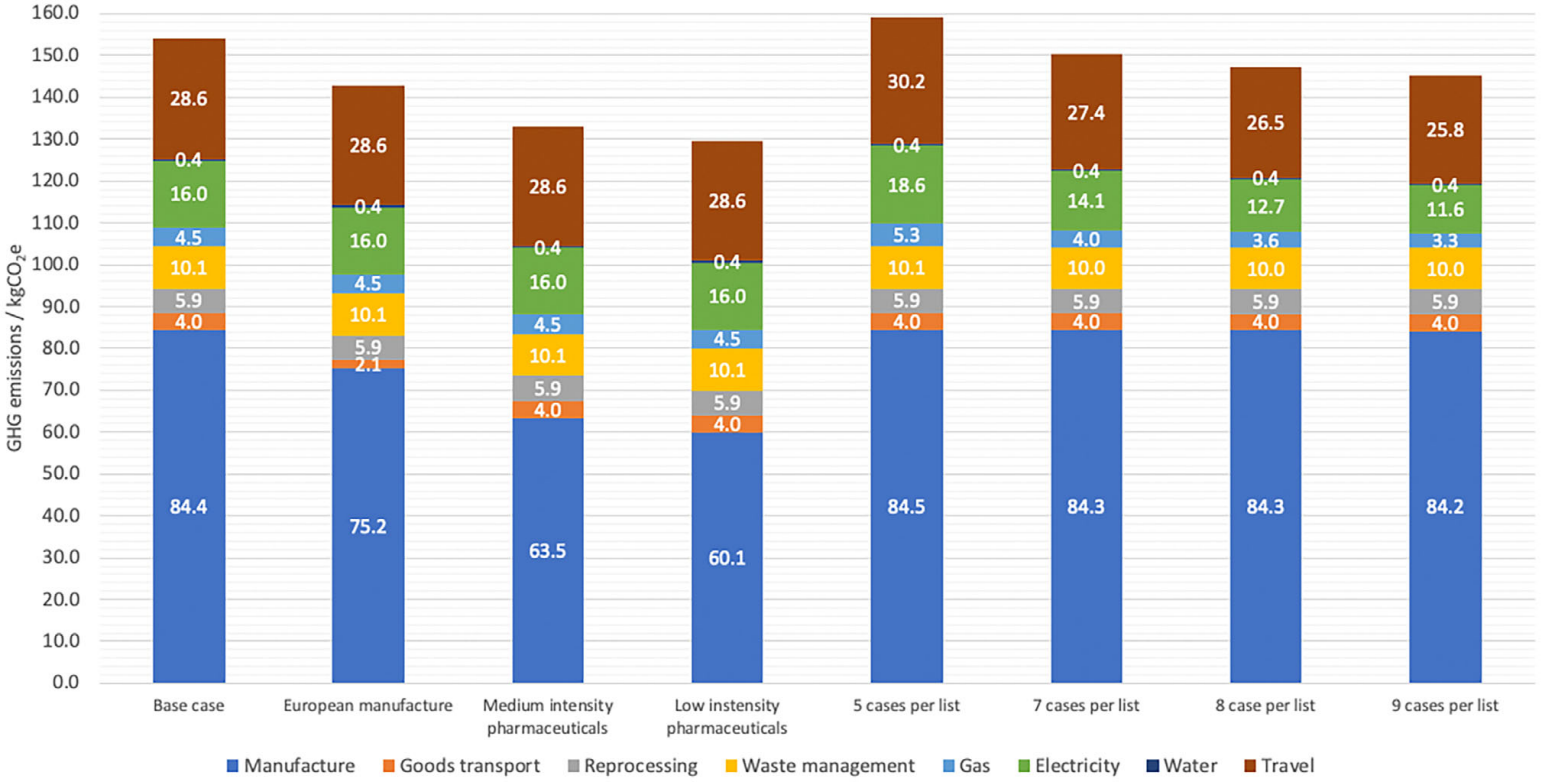
Mean GHG emissions by life‐cycle stage and relevant emissions category for the perioperative TURBT pathway, including sensitivity analysis. Intensity of pharmaceutical relates to estimated GHG emissions intensity associated with manufacture.

Figure 4 shows the different GHG emissions sources for the average perioperative TURBT pathway. In all, 57% of GHG emissions are related to the manufacture of goods and their transport to the UK. Figure 4 also shows the results of a sensitivity analysis comparing the modelled base case against different assumptions. Alternative scenarios are: manufacture of scope three products in Europe with transport by sea from Hamburg, medium and low intensity synthesis of pharmaceutical products for which specific emissions factors are not published, and theatre utilisation scenarios of between five and nine patients per operating list.

The median case GHG emissions were 131.8 kgCO_2_e. Table 2 outlines different surgical practices for which opportunities exist to routinely reduce or modify their use. Each of these individually contributes a small proportion of the overall GHG emissions associated with a perioperative TURBT pathway, but when combined and scaled across routine practice can result in large associated GHG emissions reductions. A readily realisable series of GHG emissions reduction measures for a given patient undergoing TURBT might include: same day preoperative assessment (−7.0 kgCO_2_e), rationalising personal protective equipment for patient transfers (−0.87 kgCO_2_e), avoiding three‐way catheterisation (−3.0 kgCO_2_e) and postoperative irrigation (two bags = 3.0 kgCO_2_e), using cystoscopy equipment that has been consolidated onto the resection tray without increasing the tray size (−0.81 kgCO_2_e), avoiding Ellik use (−0.58 kgCO_2_e) and bottled saline to fill the Ellik (−0.47 kgCO_2_e), and using a bucket to collect irrigation rather than suction liners (−2.02 kgCO_2_e if two liners were avoided). These changes would reduce the associated GHG emissions by 13.4% (17.7 kgCO_2_e) compared with GHG emissions due to the median case.

**Table 2:** Modifiable sources of GHG emissions related to surgical practice and suggested mitigation measures.

Items	GHG emissions per use, kgCO_2_e	Mitigation measures
Three‐way catheter equipment (22‐F catheter, 10‐mL water syringe, irrigation catheter bag)	3.00	No catheter as default. Three‐way catheter if complete haemostasis not possible
3‐L irrigation bag	1.51	Limit irrigation flow rate intraoperatively. No postoperative irrigation as default
Two‐way catheter equipment (16‐F catheter, 10‐mL water syringe, standard catheter bag)	1.68	No catheter as default. Two‐way catheter if prior long‐term catheter, or high risk of acute urinary retention or bladder perforation/deep resection
Standard catheter bag	1.35	If patient planned for home with two‐way catheter connect direct to leg bag in theatre to avoid using standard catheter bag
Catheter leg bag + straps	1.16	Attach this in theatre instead of a bedside catheter bag for patients planned for discharge with a two‐way catheter
Cystoscopy set	0.81	Consolidate equipment onto resection set to avoid additional reprocessing emissions
Biopsy forceps	1.09	Open only when indicated
Clutton sounds	0.55	Open only when indicated
Ellik evacuator	0.58	Open only when indicated
1‐L saline bottle (to fill Ellik)	0.47	Avoid use by filling Ellik with irrigation solution
2 suction liners + connection hosing	2.02	Use bucket for small resections and bucket or ‘direct to drain’ irrigation extraction for larger resections
Irrigation bucket	0.00025	Use this instead of suction liners for small resections
Mitomycin C toxic waste bin	5.25	Combine waste from two to three patients treatment into one container to reduce the number of waste bins incinerated

## Discussion

The carbon footprint of the perioperative TURBT pathway modelled in this study is broadly in line with findings from other studies across surgery [19]. Our mean perioperative TURBT pathway produced an estimated 2.4 times more GHG emissions than a prostate MRI and biopsy pathway modelled in the United States for which UK electricity emissions factors were substituted [20]. Owing to differences in time, location, boundary conditions and modelled assumptions, one can expect to find variation in the reported findings in LCAs. Our primary focus was to identify substantial GHG emissions hotspots that can be mitigated through a combination of practice change and innovation. This pathway‐based approach has achieved this aim. We have demonstrated a significant range in the GHG emissions associated with individual cases, which justifies determining actual resource used in patients’ care rather than purely modelling assumed use. By assessing the resource used to provide care for 30 patients we have obtained a reasonable reflection of practice in our unit; however, this may differ for other units depending on their practices.

Our cradle‐to‐grave LCA has identified four main GHG emission category areas which comprise 81% of average emissions for the 30 cases studies. These four categories are surgical equipment, patient and staff travel, gas and electricity, and anaesthesia, drugs and associated equipment. Within those categories, 87% of emissions are due to consumable (single use) items of processes.

A major surgical equipment hotspot was single‐use surgical field items. Single‐use TUR drapes are almost ubiquitous in endourology; however, reusable surgical drapes that are water repellent and which can be sterilised do exist. Reusable surgical drapes have been shown to have a carbon footprint 200–300% lower per use than single‐use alternatives in prior studies, with no identified impact on the rate of surgical site infection evidenced in the available literature [21, 22]. In a recent study that modelled the per‐use GHG emissions of hospital laundry, reusable items had a substantially lower carbon footprint than single‐use alternatives even when transported large distances [12]. This relates to the very high GHG emissions associated with the manufacture and incineration of single‐use synthetic textiles.

A further surgical hotspot was the delivery and extraction of irrigation. This again is driven by the life cycle emissions of single‐use plastics used to hold irrigation and to dispose of it in the case of suction liners. Urologists should aim to limit the flow of irrigation to the minimum required to perform safe and expedient surgery, and also to achieve good haemostasis at the end of each case to avoid the need for postoperative irrigation, catheterisation, and the need for inpatient admission wherever possible. Peer‐reviewed data and data available through the NHS Model Health System website indicate the feasibility of performing day case TURBT with high rates, which will inherently avoid ongoing irrigation along with the associated resources of an inpatient admission [23, 24].

Approximately 3.5% of UK road travel is related to the NHS [25]. An intuitive means of reducing travel related GHG emissions modelled in this pathway would be to promote same‐day preoperative assessment for patients identified as needing surgery. No patients underwent same‐day assessment in our study, and this is a service improvement that would offer the co‐benefits of cost and convenience to patients whilst reducing travel. In the Netherlands, digital preoperative patient assessment has been shown to have no impact on preoperative patient anxiety or postoperative recovery, whilst offering more flexible access to preoperative instruction and reducing travel [26]. Developing remote and safe care models will be fundamental in achieving the transition towards net zero and is promoted by the Getting It Right First Time and Greener NHS programmes in the UK [27, 28]. The large contribution from travel‐related GHG emissions will also reduce if we are able to continue to transition towards lower carbon forms of travel both societally and within the NHS [25].

Table 2 outlines a number of ways in which clinical decision making and operating room setup can provide opportunities to reduce resource use. Each of these changes results in a small but worthwhile GHG emissions reduction because when combined and scaled across a health system these changes have a large effect. We have provided an example showing how 17.7 kgCO_2_e of associated GHG emissions might readily be removed from one case. If this quantity could be removed from each of the 23 000 annual perioperative TURBT pathways delivered in England this would amount to 409 tonnesCO_2_e. This is equivalent to the average annual GHG emissions of 60 global citizens [29]. Alongside these sorts of mitigation measures delivered during clinical care, we require simultaneous decarbonisation measures across NHS support services such as energy supply, supply chain, construction and transport. Action taken in tandem across the NHS by the array of stakeholders responsible for these different services is necessary to achieve meaningful progress towards net zero [28].

There are further strategies that are likely to reduce the environmental impact of bladder cancer care. Compared with TURBT, flexible cystoscopy and laser ablation of selected recurrent tumours can be delivered in a low acuity clinic setting with less energy use than operating theatres, and with less equipment and fewer staff [7, 28]. Pedersen et al. [30] showed flexible cystoscopy and laser tumour ablation to be non‐inferior compared with TURBT for selected recurrent low‐grade bladder cancers over a short follow‐up period. It was also preferred by patients. This procedure therefore warrants comparison of its environmental impact against TURBT in the context of long‐term oncological management.

Overall, the sensitivity analysis revealed modest variation in estimates when different assumptions were tested. It also provides further guidance as to how the GHG emissions per operation might be further reduced. Of the assumptions, the intensity of active pharmaceutical ingredient synthesis had the largest overall effect. There is currently a relative lack of data to accurately describe the environmental impact of industrial scale manufacture of specific pharmaceutical products, with gross industry average data being a best estimate for most products [16, 31]. Further research in this area is necessary to guide mitigation steps and product choice. Modelling different theatre utilisation scenarios from five patients per list through to nine per list resulted in a modest difference in GHG emissions attributable to each case. There is a demonstrable environmental benefit to optimally filling operating theatre lists alongside the fact that this represents appropriate utilisation of expensive healthcare resource.

### Limitations

As for any environmental impact assessment, this study is limited by the fact that it is both time and place specific, and inherently involves modelling assumptions and a choice of system boundaries [5]. As such, the results should be generalised with caution and involve close inspection of our assumptions, inventory analysis, and selection of emissions factors. We have presented our results in a transparent manner to facilitate this. Our aim was to identify GHG emissions hotspots. Whilst we have achieved this, firm conclusions cannot be made about all of the modelled outputs. For example, further detailed comparison of different irrigation extraction methods is necessary across a range of volume and time scenarios, as is comparison of different TURBT anaesthetic options. We have measured practice at a single hospital and certain GHG emissions hotspots might differ at other hospitals according to local practices. One should therefore assume that our findings do not provide an exhaustive list of the modifiable GHG emissions hotspots for other sites. Whilst it is infeasible for all units to perform cradle‐to‐grave LCAs of their clinical services, local assessment to determine excessive resource use and opportunities for environmental impact reductions based on established themes is recommended [7].

We modelled gas and electricity use according to floor surface area. We did not have specific submeter readings available for operating theatres and other relevant environments. MacNeill et al. [6] demonstrated a three‐ to six‐fold increased carbon intensity of operating theatres compared to other hospital areas in three hospitals in the UK, United States, and Canada. As such, we might have underestimated energy use particularly in operating theatres. We did not model coolant use in this assessment due to data inaccessibility. There is a case for not doing so given geographic differences in the use of air conditioning; however, our overall result and some of the modelled differences in the sensitivity analysis will be underestimates as a result of this [32]. We did not measure operative time or the overall time in different clinical settings. Doing so would have facilitated a more accurate estimation of the attributable GHG emissions for each case, although would have been unlikely to alter the average hotspot magnitude.

## Conclusion

This process‐based LCA of GHG emissions for the perioperative TURBT pathway provides evidence of multiple GHG emissions hotspots and evidence of many ways in which the carbon footprint of this operation can be reduced. Key mitigation themes include minimising avoidable patient travel, rationalising equipment use, optimally filling operating theatre lists, and safely avoiding postoperative catheterisation and hospital admission where possible. A crucial next step is to design and deliver an implementation strategy for the environmentally sustainable changes demonstrated herein.

## Disclosure of Interests

No conflicts of interest exist for any of the authors of this study.

## Supporting information


**Fig. S1.** System boundaries.
**Fig. S2.** (a) Heat map showing emissions hotspots per case. (b) Re‐calibrated heat map showing emissions hotspots per case with irrigation solution exluded.
**Table S1.** Inventory of materials.
**Table S2.** Emissions factors.
**Table S3.** Staff inventory.
**Table S4.** Travel data.
**Table S5.** (a–c) Electricity emissions by area.
**Table S6.** (a, b) Gas emissions by area.
**Table S7.** (a, b) Water related emissions by area.
**Table S8.** (a, b) HSDU inventory and reprocessing emissions.
